# Rapid progression of prostate cancer in men with a *BRCA2* mutation

**DOI:** 10.1038/sj.bjc.6604453

**Published:** 2008-06-24

**Authors:** S A Narod, S Neuhausen, G Vichodez, S Armel, H T Lynch, P Ghadirian, S Cummings, O Olopade, D Stoppa-Lyonnet, F Couch, T Wagner, E Warner, W D Foulkes, H Saal, J Weitzel, A Tulman, A Poll, R Nam, P Sun, the Hereditary Breast Cancer Study Group

**Affiliations:** 1 Women's College Research Institute, 790 Bay Street, 7th Floor, Women's College Hospital, University of Toronto, Toronto, Ontario, Canada; 2 Department of Epidemiology, University of California, Irvine, USA; 3 University Health Network, Toronto, Ontario, Canada; 4 Department of Preventive Medicine and Public Health, Creighton University School of Medicine, Omaha, NE, USA; 5 Epidemiology Research Unit, Research Centre, Centre Hospitalier de l'Universitaire Montréal, CHUM Hôtel Dieu, Montreal, Quebec, Canada; 6 Chicago Center for Clinical Cancer Genetics, University of Chicago, Chicago, IL, USA; 7 Institut Curie, Paris, France; 8 Mayo Clinic, Rochester, MN, USA; 9 Department of Gynecology, Division of Senology, Medical University of Vienna and Private Trust for Breast Health, Vienna, Austria; 10 Sunnybrook Regional Cancer Center, Toronto, Ontario, Canada; 11 Departments of Medicine and Oncology, McGill University, Montreal, Quebec, Canada; 12 Hereditary Cancer Program, Division of Human Genetics, Children's Hospital Medical Center, Cincinnati, OH, USA; 13 City of Hope Department of Cancer Genetics, City of Hope National Medical Center, Duarte, CA, USA

**Keywords:** prostate cancer, *BRCA1*, *BRCA2*

## Abstract

Men with *BRCA2* mutations have been found to be at increased risk of developing prostate cancer. There is a recent report that *BRCA2* carriers with prostate cancer have poorer survival than noncarrier prostate cancer patients. In this study, we compared survival of men with a *BRCA2* mutation and prostate cancer with that of men with a *BRCA1* mutation and prostate cancer. We obtained the age at diagnosis, age at death or current age from 182 men with prostate cancer from families with a *BRCA2* mutation and from 119 men with prostate cancer from families with a *BRCA1* mutation. The median survival from diagnosis was 4.0 years for men with a *BRCA2* mutation *vs* 8.0 years for men with a *BRCA1* mutation, and the difference was highly significant (*P*<0.01). It may be important to develop targeted chemotherapies to treat prostate cancer in men with a *BRCA2* mutation.

*BRCA2* is a multisite cancer gene. It is generally thought that *BRCA2* mutations primarily affect women, but men with mutations are also at elevated cancer risk. The two most important cancer sites for males who carry a mutation are the prostate and the pancreas (Liede *et al*, 2004). The risk of prostate cancer is elevated approximately fivefold in *BRCA2* carriers, compared to noncarriers. Genetic counselors and urologists advise men with *BRCA2* mutations to undergo surveillance with annual PSA testing from the age of 40 years – a recommendation based on the perceived effectiveness of prostate screening. It is hoped that screening leads to early diagnosis, when cure rates are high. A recent study from Iceland suggests that prostate cancers in men with a *BRCA2* mutation may be unusually aggressive (Tryggvadottir *et al*., 2007). Tryggvadottir *et al* (2007) identified the Icelandic founder mutation (*BRCA2* 999 del5) in 30 of the 527 prostate cancer patients studied (5.7%). Men with a *BRCA2* mutation had a median survival of only 2.1 years, compared with 12.4 years for noncarriers (*P*<0.01). The survival difference could not be explained by stage or grade. It is important that these findings be replicated because of the implications for the screening of men with a *BRCA2* mutation. We identified the prostate cancer patients in a panel of 2673 families with a *BRCA1* or a *BRCA2* mutation and estimated survival of the men in the two subgroups.

## Methods

Men with prostate cancer were included in the survival analysis if they were from a family with a BRCA mutation and if they were (a) known to carry the familial BRCA mutation, or (b) if they were a first-degree relative of a known carrier, or (c) if they were a first-degree relative of a woman diagnosed with breast or ovarian cancer. For each eligible man with prostate cancer, information was collected on age at diagnosis, age at death (if deceased) or age when last known alive (if alive). Information was collected by the review of the family pedigree and the medical record of the proband. In some cases, this was supplemented by the interview of another family member. Men who had been diagnosed with other forms of cancer were excluded.

We identified 938 families with a *BRCA2* mutation in the database. These families came from 33 different clinical centres in five countries. Of these, 277 families contained one or more cases of prostate cancer (29.5%). In aggregate, the 277 families included 434 men with prostate cancer (mean 1.6 per family). In all 141 of the cases were ineligible (either known to be a noncarrier, married in, not closely related to a carrier or an affected woman or had a history of cancer other than prostate). Of the remaining 293 prostate cancer patients, we obtained data on age at diagnosis and age at death (or current age if alive) on 183 (62%). Of these, 67 were determined to carry the familial *BRCA2* mutation and 116 men were probable mutation carriers.

Men from families with *BRCA1* mutations were selected as the comparison group. To ensure comparability, we used the same inclusion criteria as were used for the *BRCA2* carriers. We identified 1735 families with a *BRCA1* mutation, of which 316 families contained one or more cases of prostate cancer (18.2%). The 316 families included 457 men with prostate cancer (a mean of 1.4 per family). A total of 252 of the cases were ineligible (either known to be a noncarrier, married in, not closely related to a carrier or an affected woman or had a history of cancer other than prostate); of the remaining 205 patients, we had data on age at diagnosis and age at death (or current age if alive) on 119 men (58%). Of these, 37 were determined to carry the familial *BRCA1* mutation and 82 men were likely to be a carrier.

We performed survival analysis to establish the overall survival of *BRCA2* mutation carriers with prostate cancer and the relative survival as compared to *BRCA1* carriers. Patients were followed from the year of diagnosis until year of death (if deceased) or year when last known alive (in most cases 2007). Kaplan–Meier methods were used to construct survival curves, and the significance of the comparison of the curves was based on the log-rank test. A hazard ratio was estimated using the Cox proportional hazards model implemented in SAS version 9.1.3 version The hazard ratio was adjusted for age of diagnosis.

## Results

**Figure 1:**
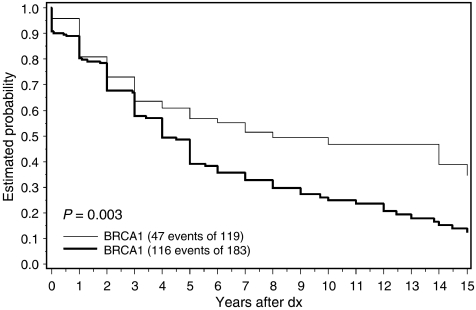
Probability of survival after prostate cancer in men from families with *BRCA1* and *BRCA2* mutations (all causes of death).

There were 183 men with prostate cancer who were known or probable carriers of a *BRCA2* mutation and 119 men with prostate cancer who were known or probable carriers of a *BRCA1* mutation. The average age of diagnosis was similar for men in the two groups (Table 1). The median survival time was 8.0 years for *BRCA1* carriers (or probable carriers) and was 4.0 years for *BRCA2* carriers (or probable carriers) (Figure 1). The 5-year overall survival was 57% for *BRCA1* carriers and was 39% for *BRCA2* carriers. The 10-year survival was 47% for *BRCA1* carriers and 25% for *BRCA2* carriers. The 15-year overall survival was 35% for *BRCA1* carriers and 12% for *BRCA2* carriers. We estimated the hazard ratio for all-cause mortality for *BRCA2 vs BRCA1* carriers, adjusted for age, over the entire follow-up period. We found that the risk of dying in cases from BRCA2 families was 70% higher than in cases from BRCA1 families (HR=1.7; 95% CI 1.2–2.4) (Table 2).

**Figure 2:**
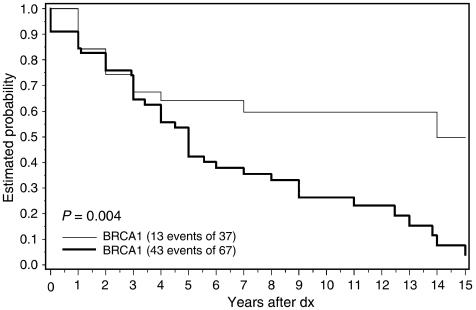
Probability of survival after prostate cancer in known carriers with *BRCA1* and *BRCA2* (all causes of death).

The analysis was then repeated, including only known carriers (67 *BRCA2* and 37 *BRCA1*). The median survival time was 15 years for carriers of a *BRCA1* mutation and was 5.0 years for carriers of a *BRCA2* mutation (Figure 2). The 5-year overall survival was 64% for *BRCA1* carriers and 42% for *BRCA2* carriers. The 10-year survival was 60% for *BRCA1* carriers and 26% for *BRCA2* carriers. The 15-year overall survival was 50% for *BRCA1* carriers and 4% for *BRCA2* carriers. We estimated the hazard ratio for all-cause mortality for *BRCA2 vs BRCA1* carriers, adjusted for age, over the entire follow-up period. The hazard ratio was 2.5 (95% CI 1.3–4.7; *P*=0.005).

## Discussion

In this study, we examined survival for men with prostate cancer and a *BRCA2* mutation, and compared this to a similar group of men with a *BRCA1* mutation. Prostate cancer is a clinical manifestation of the *BRCA2* gene, but only a small proportion of prostate cancers are attributable to *BRCA2* mutations. An excess risk of prostate cancer among men with *BRCA2* mutations has been previously documented. Epidemiology studies have been of two types: (1) estimating the risk of prostate cancer in men from families with *BRCA2* mutations, and (2) estimating the proportion of *BRCA2* mutations among unselected men with prostate cancer. In a study of 173 families with *BRCA2* mutations, the Breast Cancer Linkage Consortium reported in 1999 that there was a significantly increased risk of prostate cancer among male first-degree relatives of female carriers (Breast Cancer Linkage Consortium, 1999). They estimated the odds ratio to be 4.7 (95% CI 3.5–56.2). In a study in the Netherlands of 139 families with *BRCA2* mutations, Van Asperen *et al* (2005) confirmed an excess risk of prostate cancer in first-degree relatives of carriers (OR=2.5; 95% CI 1.6–3.8). Given that only one-half of the first-degree relatives are expected to be gene carriers, this corresponds to a relative risk of prostate cancer, given a *BRCA2* mutation, of approximately five.

Kirchoff *et al* (2004) tested 251 unselected Ashkenazi Jewish men with prostate cancer from the New York area for the two founder mutations in *BRCA1* and the one in *BRCA2.* They also tested 1472 Ashkenazi controls. A mutation was found in 5.2% of cases and 1.9% of controls. The presence of a *BRCA2* mutation was associated with a 4.8-fold increased risk of prostate cancer. In contrast, the *BRCA1* carriers in that study were not at increased risk of prostate cancer. In a study from the United States, Agalliu *et al* (2007) found a *BRCA2* mutation in two of 290 men diagnosed with prostate cancer under the age of 55 (mixed ethnicities). The relative risk for early-onset prostate cancer was 7.8.

We observed that men with prostate cancer and a *BRCA2* mutation experienced relatively poor survival, in comparison to men with prostate cancer and a *BRCA1* mutation. The median survival for *BRCA2* carriers was 4.0 years, and at 10 years post-diagnosis, 53% of the patients had died.

There are several limitations to our study. Ideally, one would follow a cohort of unselected patients with prostate cancer and a *BRCA2* mutation, record details on the stage, grade and all treatments received, and then compare the outcome of the hereditary group with a similar group of patients without a mutation. We do not know the cause of death for these patients and expect that some will have died of causes other than prostate cancer. Not all of the patients in our study were proven carriers of a *BRCA2* mutation; we included 67 carriers (or obligate carriers) and 116 men with prostate cancer who had not been tested for the presence of the mutation. The families in this study were those referred to various cancer genetics centres because of multiple cases of breast and/or ovarian cancer in the family, and were not selected on the basis of prostate cancer. The demographic and clinical information on the patients with prostate cancer were taken from a pedigree review and interview of the proband; we did not review the medical record or the pathology report of the prostate cancer patient. The ages of diagnosis and death were based on information provided by the female proband. It may be probable that not all cases of prostate cancer in male relatives were recorded. We do not know if the case was diagnosed clinically or through prostate cancer screening. We do not have information on the stage or grade at presentation, and we did not record details of treatment.

Despite these numerous limitations, we believe that the differences observed in survival of the *BRCA1* and *BRCA2* carriers (Figures 1 and 2) can be attributed to the adverse effect of the *BRCA2* mutation on prostate cancer survival. These data were recorded and collected using the same methods for the *BRCA1* and *BRCA2* families. There is no reason to believe that carriers of *BRCA2* mutations should have greater mortality than men with *BRCA1* mutations from non-cancer causes, and men with other forms of cancer were excluded. The survival experience of the *BRCA2* carriers is almost universally poor, as only 26% of the known carriers were alive 10 years after diagnosis and 4% were alive at 15 years. Compared to men with a *BRCA1* mutation, men with a *BRCA2* mutation in this study were more than two times as likely to die (of any cause) following the diagnosis of prostate cancer (hazard ratio 2.5), and it is likely that the relative mortality for prostate cancer-specific mortality would be even more extreme. These data support the conclusions of Tryggvadottir *et al* (2007) who reported that men with prostate cancer and a *BRCA2* mutation experienced an unexpectedly high rate of mortality. The basis for the aggressive behaviour of the BRCA2-associated prostate cancers is not known. Moro *et al* (2008) show that downregulation of BRCA2 expression through the introduction of siRNA in prostate cancer cells promoted cancer cell migration and invasion. Mitra *et al* (2008) found the average grade of prostate cancers among men with BRCA2-associated prostate cancers (Gleason score) to be higher than that of noncarrier control tumours.

We compared the survival of men with *BRCA2* mutations with that of men with *BRCA1* mutations. Ideally, we would have included a comparison group of noncarriers as well. However, it was not possible to identify a similar group of noncarrier patients from this database, given that we routinely collect pedigree information only on families with a mutation in one of the two genes. Although we do not have a comparable group of noncarriers in this study, it has been reported in a trial of patients undergoing watchful waiting *vs* prostatectomy that 27% of men with prostatectomy died within 10 years of diagnosis (all-cause mortality) and 32% of men with watchful waiting died within 10 years of diagnosis (Bill-Axelson *et al*, 2005). In comparison, 75% of the BRCA2 carriers in our study died within 10 years of diagnosis.

If survival in *BRCA1* carriers is atypical, then our observed difference will not be representative of prostate cancer in general. To our knowledge, *BRCA1* is not associated with an improved survival experience. Furthermore, the risk of prostate cancer in *BRCA1* carriers is increased only to a small extent, if any (Kirchoff *et al*, 2004; Cybulski *et al*, 2008), and an increased risk is not associated with all mutations (Cybulski *et al*, 2008). Therefore, we expect the survival experience of men with prostate cancer in *BRCA1* families to be similar to that of the general population.

The data from the two studies suggest that men with *BRCA2* mutations may not benefit from current therapies to the same extent as other men. We do not know which men in this cohort had screen-detected prostate cancer, and it will be important to study whether or not men who have subclinical, screen-detected prostate cancer also experience a high mortality rate. To address this issue, we plan to identify the *BRCA2* carriers among a large sample of prostate cancer patients and to characterise them in terms of clinical presentation, pathology and response to treatment. Future studies should also address whether current surgical and non-surgical treatments improve survival from prostate cancer among *BRCA2* carriers. Current treatments include surgery, hormonal-based therapies and radiotherapy. Given that the *BRCA2* protein is involved in the repair of damaged DNA breaks, and that radiation induces double-strand breaks, it is possible that prostate cancer patients with a *BRCA2* mutation are more sensitive to radiotherapy than patients without a mutation. Cytotoxic chemotherapy is not generally used in the early treatment of prostate cancer. Currently, there is interest in identifying directed treatments for breast cancer in *BRCA1* and *BRCA2* carriers. Drugs that induce DNA-strand breaks, such as cis-platinum, show increased sensitivity in pre-clinical models, and trials are now underway to study cis-platinum and PARP1 inhibitors in women with breast cancer and a BRCA mutation. A male patient with metastatic prostate cancer and a *BRCA2* mutation has responded well to a PARP1 inhibitor (A Tutt, J De Bono, personal communication). It will be a matter of considerable interest to see whether or not the men with prostate cancer and a *BRCA2* mutation benefit from targeted chemotherapy.

## Figures and Tables

**Table 1:** Characteristics of study subjects

	**BRCA1**	**BRCA2**	
**Variables**	***N*=119 (37 known carriers)**	***N*=183 (67 known carriers)**	***P***
*Age at diagnosis (range)*			
All subjects	66.9 (41–86)	67.1 (32–97)	0.87
Known carriers	70.2 (55–86)	67.1 (49–84)	0.08
			
*Vital status N (%)*			
All subjects			
Alive	72 (60.5)	67 (36.6)	
Dead	47 (39.5)	116 (63.4)	<0.0001
			
*Known carriers*			
Alive	24 (64.9)	24 (35.8)	
Dead	13 (35.1)	43 (64.2)	0.004
			
*Mean follow-up years (range)*			
All subjects	4.3 (0–15)	4.1 (0–15)	0.74
Known carriers	5.7 (0–15)	4.3 (0–10)	0.14

*P*-values calculated for mean using Student's *t*-test, and for frequencies using *χ*^2^ test.

**Table 2:** All cause mortality in *BRCA2* *vs* *BRCA1* patients with prostate cancer

	**Univariate**		**Age-adjusted**	
	**HR (95% CI)**	***P***	**HR (95% CI)**	***P***
*BRCA2* *vs* *BRCA1* All subjects	1.64 (1.17–2.30)	0.005	1.71 (1.22–2.41)	0.002
*BRCA2* *vs* *BRCA1* Known carriers	2.41 (1.28–4.52)	0.006	2.48 (1.31–4.70)	0.005

Hazard ratio represents mortality experience of patients with BRCA2 mutations compared to those with BRCA1 mutations, calculated using Cox proportional hazards model.
